# Clustering under the line graph transformation: application to reaction network

**DOI:** 10.1186/1471-2105-5-207

**Published:** 2004-12-24

**Authors:** Jose C Nacher, Nobuhisa Ueda, Takuji Yamada, Minoru Kanehisa, Tatsuya Akutsu

**Affiliations:** 1 Bioinformatics Center, Institute for Chemical Research, Kyoto University, Uji, 611-0011, Japan

## Abstract

**Background:**

Many real networks can be understood as two complementary networks with two kind of nodes. This is the case of metabolic networks where the first network has chemical compounds as nodes and the second one has nodes as reactions. In general, the second network may be related to the first one by a technique called line graph transformation (i.e., edges in an initial network are transformed into nodes). Recently, the main topological properties of the metabolic networks have been properly described by means of a hierarchical model. While the chemical compound network has been classified as hierarchical network, a detailed study of the chemical reaction network had not been carried out.

**Results:**

We have applied the line graph transformation to a hierarchical network and the degree-dependent clustering coefficient *C*(*k*) is calculated for the transformed network. *C*(*k*) indicates the probability that two nearest neighbours of a vertex of degree *k *are connected to each other. While *C*(*k*) follows the scaling law *C*(*k*) ~ *k*^-1.1 ^for the initial hierarchical network, *C*(*k*) scales weakly as *k*^0.08 ^for the transformed network. This theoretical prediction was compared with the experimental data of chemical reactions from the KEGG database finding a good agreement.

**Conclusions:**

The weak scaling found for the transformed network indicates that the reaction network can be identified as a degree-independent clustering network. By using this result, the hierarchical classification of the reaction network is discussed.

## Background

Recent studies on network science demonstrate that cellular networks are described by universal features, which are also present in non-biological complex systems, as for example social networks or *WWW*. Most networks encountered in real world have scale-free topology, in particular networks of fundamental elements of cells as proteins and chemical substrates [1-4]. In these networks, the distribution of node degree follows a power-law as *P*(*k*) ~ *k*^-*γ *^(i.e., frequency of the nodes that are connected to *k *other nodes). The degree of a node is the number of other nodes to which it is connected.

One of the most successful models for explaining that scale-free topology was proposed by *Barabási-Albert *[5], which introduced a mean-field method to simulate the growth dynamics of individual nodes in a *continuum theory *framework. However, although that model was a milestone to understand the behavior of real complex networks, it could not reproduce all the observed features in real networks such as clustering dependence. The observed properties of networks with *N *nodes are: scale-free of degree distribution *P*(*k*) ~ *k*^-*γ*^, power-law scaling of clustering coefficient *C*(*k*) ~ *k*^-1 ^and a high value for the average of the clustering coefficient <*C *> and its independence with network size. In particular, the dependence of *C*(*k*) ~ *k*^-1 ^was one of the results obtained by [6]. In order to bring under a single framework all these observed properties in real networks *Ravasz et al. *(the RSMOB model in what follows) suggested successfully a hierarchical and modular topology [7,8]. In [8], a network with the above mentioned properties was called hierarchical network. We note that this deterministic model is an extension of the original model shown in [9]. It is also worth noticing that this modular topology was also suggested in biological networks by [10,11]. Interestingly, these properties of networks have been found in many non- biological and biological networks. One of them, which is the subject of our study, is the metabolic network.

It is interesting to note that the metabolic network is an example of bipartite networks [12]. In a bipartite network there are two kinds of nodes and edges only connect nodes of different kinds. In the metabolic network these nodes are chemical compounds and reactions. The network generated by the chemical compounds (reactions) is called compound (reaction) projection. A line graph transformation (i.e., each edge between two nodes becomes a node of the transformed network) may relate both projections.

However, although the line graph transformation works fine on bipartite networks, the transformed network (in the particular case of metabolic networks) may not be totally the same as the reaction projection. This issue is discussed in detail later. In addition, we will show by comparing with the experimental data, that this fact does not affect our qualitative results. Furthermore, a detailed analysis of the line graph transformation focused on the degree distribution *P*(*k*) and applied to some real networks can be found in [13]. In that work, similarities and differences between the line graph transformation and the metabolic network are also discussed. There it was found that if the initial network follows a power-law *P*(*k*) ~ *k*^-*γ*^, the transformed network preserves the scale-free topology and in most cases the exponent is increased by one unit as *P*(*k*) ~ *k*^-*γ*+1^.

It is also worth noting that the line graph transformation has recently been applied with success by *Pereira-Leal et al. *[15] on the protein interaction network with the aim to detect functional modules. In that work, the edges (interactions) between two proteins become the nodes of the transformed network (interaction network). By means of the line graph transformation, the interaction network has a higher clustering coefficient than the protein network. By using the TribeMCL algorithm [16] they are able to detect clusters in the more highly clustered interaction network. These clusters are transformed back to the initial protein-protein network to identify which proteins can form functional clusters. At this point, we note that the aim of our study is not to detect functional modules from the metabolic network. In our work the line graph transformation is used successfully to evoke general topological properties related to the clustering degree of the reaction network.

The observed topological properties related to the clustering degree of the metabolic network (in particular, the chemical compound network) have been properly described by means of the RSMOB model. In the present work, our aim is to study the clustering coefficients *C*(*k*) and <*C *> of the reaction network by using two approaches: Firstly, we derive mathematical equations of those coefficients in the transformed network. Secondly, we apply the line graph transformation to a hierarchical network. The results from both methods are compared with experimental data of reactions from KEGG database [14] showing a good agreement. Though we started this work motivated by theoretical interest in the line graph transformation, the results provide explanation for the difference of *C*(*k*) between the compound network and the reaction network.

In our work, the hierarchical network is generated by the RSMOB model, where the nodes correspond to chemical compounds and the edges correspond to reactions. While the RSMOB model reproduces successfully the hierarchical properties of the compound network, here we show that this hierarchical model also stores adequate information to reproduce the experimental data of the reaction network. Our study indicates that it is enough to apply the line graph transformation to the hierarchical network to extract that information. While *C*(*k*) follows the power-law *k*^-1.1 ^for the initial hierarchical network (compound network), *C*(*k*) scales weakly as *k*^0.08 ^for the transformed network (reaction network). Consequently, we conclude that the reaction network may not be classified as a hierarchical network, as it is defined in [8].

### Remark

In [8], a network with scale-free topology, scaling law of *C*(*k*) ~ *k*^-1^, and high degree of clustering was called *hierarchical network*. Consequently, the RSMOB model shown in [7,8] was developed to bring these properties under a single roof. Furthermore, in [7,8,17] some networks (in particular metabolic network) were classified as hierarchical network according to the above definition. To be precise, it was argued that the signature of the intrinsic hierarchy (or hierarhical modularity) is the scaling law of *C*(*k*). Moreover, in a more recent work [18], it was claimed that traditional random and scale-free models do not have a hierarchical topology because *C*(*k*) is independent of *k *(i.e., flat plot of *C*(*k*)). In addition, analyses of *C*(*k*) were recently carried out in [19] to uncover the structural organization and hierarchy of non-biological weighted networks. At this point, we must note that we have followed the research done by *Barabási et al*, [7,8,18] and consequently, we have used its definition of hierarchical network in the present work.

However, it is also worth noticing that another way to quantify the hierarchical topology of a network is recently introduced by [20,21]. It is based on the concept of a *hierarchical path*: a path between nodes *i *and *j *is called hierarchical if (1) the node degrees grow monotonously ("*up path*"), and it is followed by a path where the node degrees decrease monotonously ("*down path*") or (2) the node degrees along this path changes monotonously from one node to the other. The fraction of *shortest paths *in a network, which are also *hierarchical paths *is called *H*. If *H *is very close to 1, the network shows a hierarchical organization. This definition seems interesting, and consequently, as a future work it would be worth to examine some biological networks (in particular, metabolic networks) by using this approach. One remark about this concept is that it focuses on hierarchy and may not contain enough information about modularity or clustering. For a brief discussion of these issues, we refer to some very useful notes written by *Dorogovtsev et al. *[21] (see also [22] for further information about related topics in networks).

## Results and discussion

### Clustering coefficients *C*(*k*) and <*C *>

**Figure 1:**
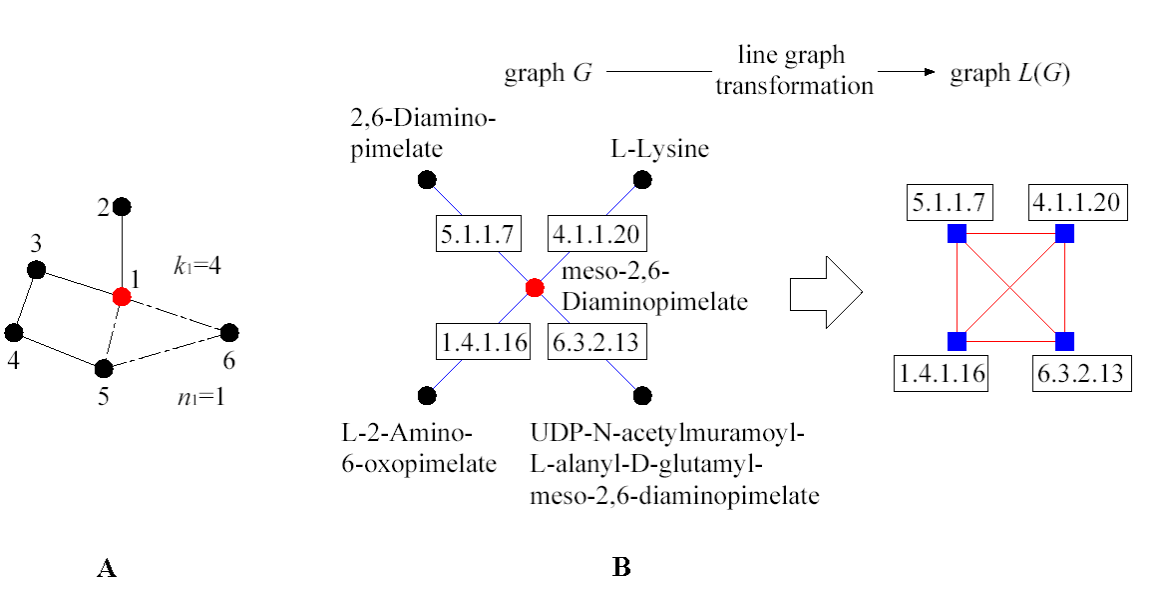
(A) Example of clustering in an undirected network. Continuous and dash-dotted lines mean interaction between nodes. In addition, the dash-dotted line defines the only triangle where the node 1 (red) is one of the vertices. The node 1 has 4 neighbors (*k*_*i *_= 4), and among these neighbors only one pair is connected (*n*_1 _= 1). The total number of possible triangles that could go through node *i *is 6. Thus, the clustering coefficient has the value *C*_1 _= 1/6. High density of triangles means high clustering coefficient. (B) We show an example of the line graph transformation. The initial graph *G *corresponds to one subgraph which belongs to the Lysine Biosynthesis metabolic pathway. This graph is constructed by taking nodes as chemical compounds and edges as reactions. By applying the line graph transformation we find graph *L*(*G*), which is the reaction graph embedded in the graph *G*. The nodes of the graph L(G) are the reactions of the graph *G *[13].

Recent analyses have demonstrated that the metabolic network has a hierarchical organization, with properties as: scale-free degree distribution *P*(*k*) ~ *k*^-*γ*^, power-law dependence of clustering coefficient *C*(*k*) ~ *k*^-1 ^and independence with network size of the average clustering coefficient <*C *>, where *N *is the total number of nodes in a network [7]. The clustering coefficient can be defined for each node *i *as:



where *n*_*i *_denotes the number of edges connecting the *k*_*i *_nearest neighbors of node *i *to each other, *C*_*i *_is equal to 1 for a node at the center of a fully interlinked cluster, and it is 0 for a node that is a part of a loosely connected cluster [7]. An example can be seen in Fig. 1A.

Geometrically, *n*_*i *_gives the number of triangles that go through node *i*. The factor *k*_*i*_(*k*_*i *_- 1)/2 gives the total number of triangles that could go through node *i *(i.e., total number of triangles obtained when all the neighbors of node *i *are connected to each other). In the case of Fig. 1A, there is one triangle that contains node 1 (dash-dotted lines), and a total of 6 triangles could be generated as the maximum. Hence, the clustering coefficient of node 1 is *C*_1 _= 1/6.

On the other hand, the average clustering coefficient <*C *> characterizes the overall tendency of nodes to form clusters as a function of the total size of the network *N*. The mathematical expression is:



The structure of the network is given by the function *C*(*k*), which is defined as the average clustering coefficient over nodes with the same node degree *k*. This function is written as:



where *N*_*k *_is the number of nodes with degree *k*, and the sum runs over the *N*_*k *_nodes with degree *k*. A scaling law *k*^-1 ^for this magnitude is an indication of the hierarchical topology of a network.

Once the theoretical definitions have been introduced, our aim is to analyse how the coefficients <*C *> and *C*(*k*) are modified under the line graph transformation.

### Line graph transformation to metabolic networks: spurious nodes

Given an undirected graph *G*, defined by a set of nodes *V*(*G*) and a set of edges *E*(*G*), we associate another graph *L*(*G*), called the line graph of *G*, in which *V*(*L*(*G*)) = *E*(*G*), and two nodes are adjacent if and only if they have a common endpoint in *G *(i.e., *E*(*L*(*G*)) = {{(*u*, *v*), (*v*, *w*)}|(*u*, *v*) ∈ *E*(*G*), (*v*, *w*) ∈ *E*(*G*)}). This construction of graph *L*(*G*) from the initial graph *G *is called line graph transformation [23].

It is worth noting that in a previous work [13] the degree distribution *P*(*k*) was studied by applying line graph transformation to synthetic and real networks. There it is assumed an initial graph *G *with scale-free topology as *P*(*k*) ≈ *k*^-*γ*^. As the degree of each transformed node (i.e., an edge in *G*) will be roughly around *k*, the distribution of the line graph *L*(*G*) should be *k*·*k*^-*γ *^= *k*^-*γ*+1 ^with degree around *k*. Therefore, it is concluded that if we have a graph *G *with a probability distribution following a power-law as *k*^-*γ*^, then *L*(*G*) will follow a power-law as *k*^-*γ*+1^. The real networks under study were protein-protein interaction, *WWW*, and metabolic networks. In Fig. 1B, we can see an example of the line graph transformation applied to a subgraph of the metabolic network.

**Figure 2:**
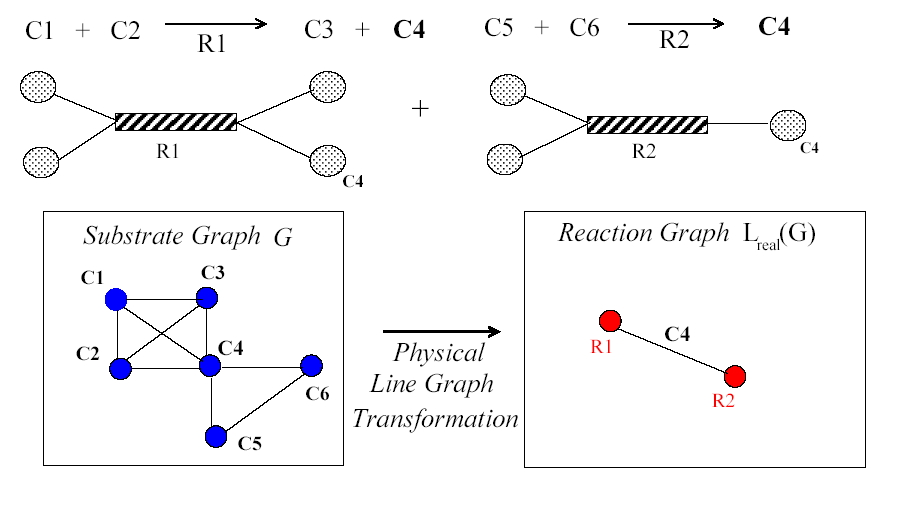
We show two reactions (*R1*, *R2*) sharing a common chemical compound, and both reactions contain more than one product (or substrate). The substrate graph *G *(chemical compounds) is shown dark blue circles. The reaction graph *L*_*real *_(reactions) is shown with light red circles. If we apply a line graph transformation to this network, we would obtain more than two nodes in the transformed network. However, only two nodes (reactions) are present in the real process. These cases are computed and transformed by generating only as many nodes in the transformed network as reactions in the real metabolic process. We call this procedure *physical *line graph transformation.

However, it is important to point out one issue. In metabolic networks, there are cases where spurious nodes appear (see Fig. 2). For example, we consider two reactions sharing the same substrate (or product) and at least one of the chemical reaction has more than one product (or substrate). If we apply a line graph transformation to this network, we would obtain more than two nodes in the transformed network, where only two nodes (reflecting two reactions present in the real process) should appear. These spurious nodes appear only when one (or some) reaction(s) in the network has more than one product (or substrate). Therefore, these cases should be computed and transformed by generating only as many nodes in the transformed network as reactions in the real metabolic process. This procedure is called *physical *line graph transformation. In the present work, we have applied this procedure to generate the reaction network by using experimental data from the KEGG database. Experimental data are shown later in Figs. 7B and 8 (blue diamonds). More detailed information about this issue can be found in [13].

### Equations of *C*(*k*) and <*C *> under the line graph transformation

**Figure 3:**
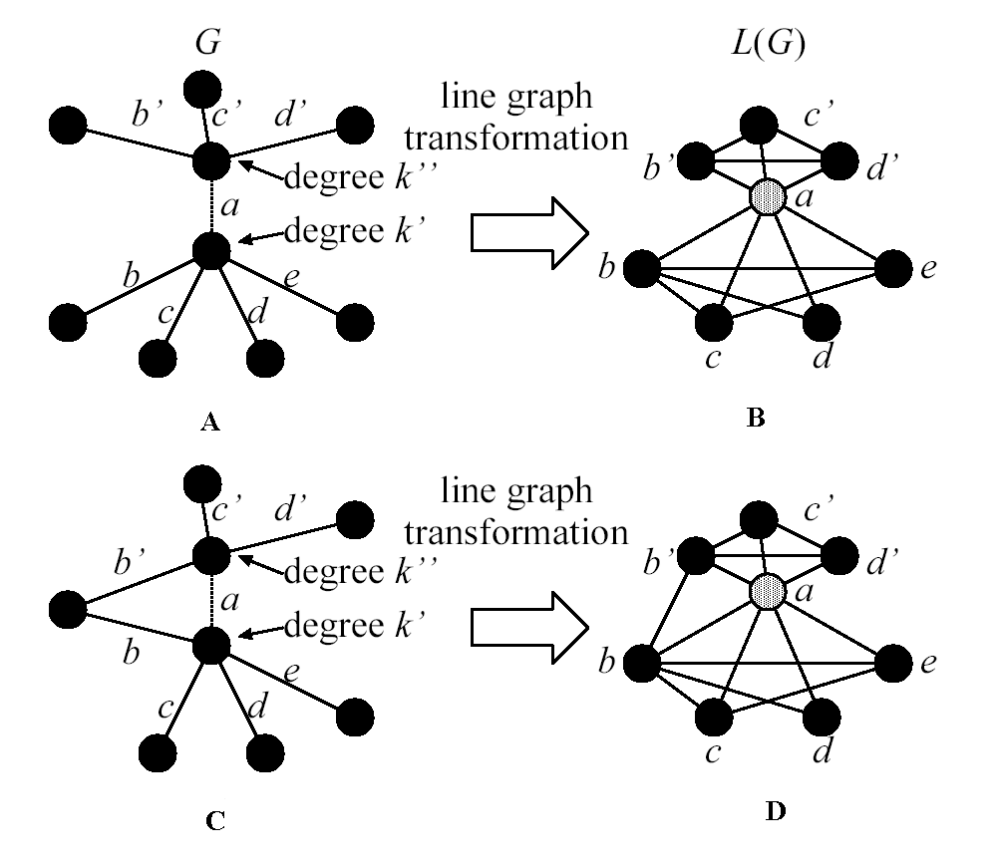
(A) Graph *G *with two hubs with degree *k*' and *k*" connected by edge *a*. (B) The corresponding line graph *L*(*G*) after the line graph transformation is done. (C) Graph *G *where edges *b *and *b*' have a common node as endpoint. (D) Line graph of (C). It is worth noticing that (D) has only one more edge than (B). Hence, (D) has one more triangle that go through node *a *than (B).

We assume a graph *G *as it is depicted in Fig. 3A. In this graph, edge *a *connects two nodes with degree *k*' and *k*". We apply the line graph transformation to this graph *G *and the result of this transformation is the line graph of *G*, *L*(*G*) shown in Fig. 3B. We see that, under the line graph transformation, the nodes of *L*(*G*) are the edges of *G*, with two nodes of *L*(*G*) adjacent whenever the corresponding edges of *G *are.

**Figure 4:**
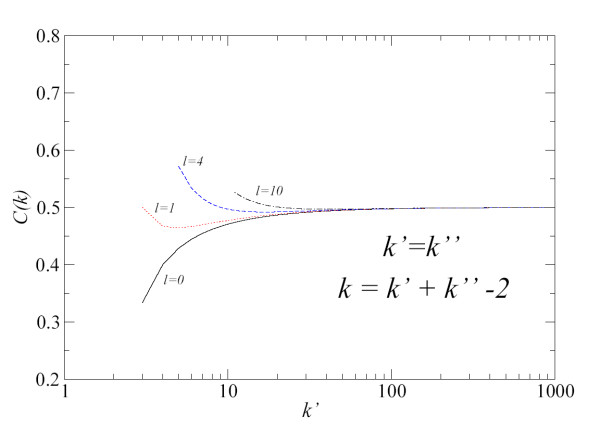
Values of *C*_*a*_(*k*) from Eq. (5) calculated by taking *k*' = *k*". Number of common nodes as endpoint of two edges (triangles) are indicated by the parameter *l*. The degree of transformed nodes is *k *= *k*' + *k*" - 2 because the edge *a *vanishes in the graph *L*(*G*).

**Figure 5:**
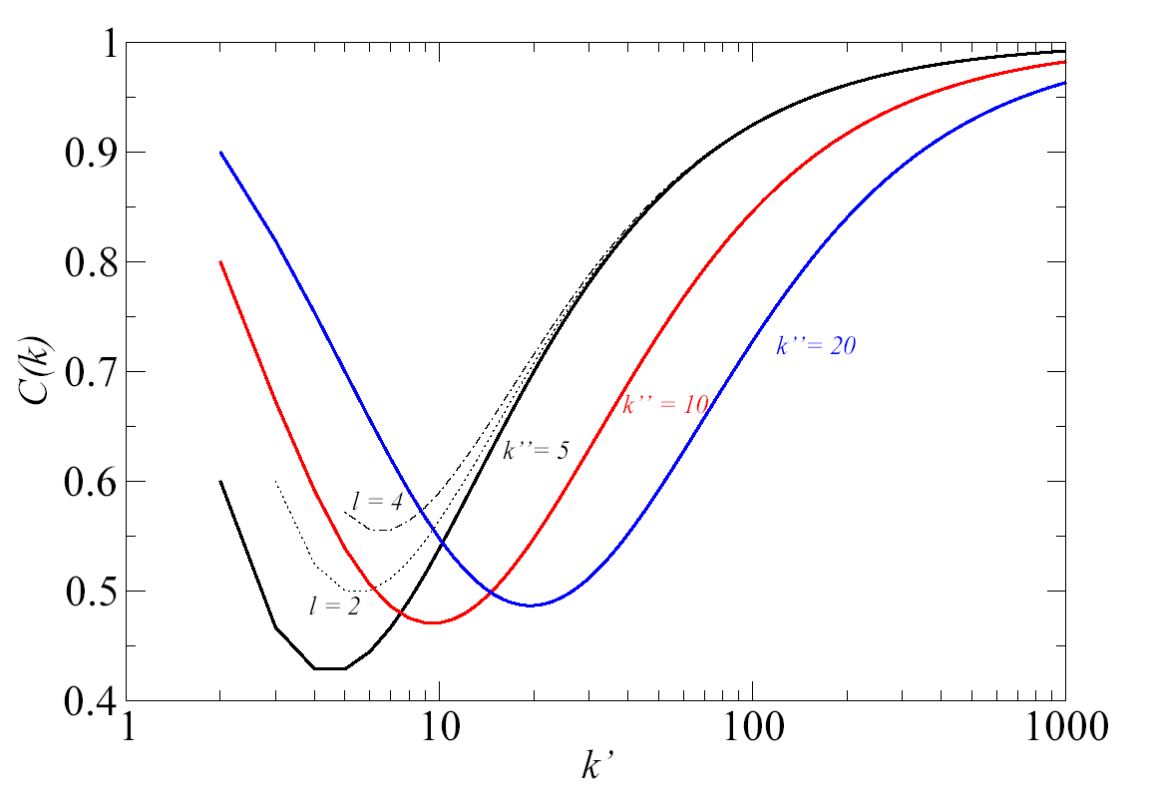
Values of *C*_*a*_(*k*) from Eq. (5) calculated by taking *k*" with constant values as *k*" = 5 (black line), *k*" = 10 (red), *k*" = 20 (blue) and *k*' as a free parameter. Dotted and dash-dotted lines show the presence of triangles (*l *≠ 0). Triangles increase the value of *C*_*a*_(*k*).



The clustering coefficient for the node *a *in the transformed network can be written by using Eq. (1) as:



where *k *= *k*' + *k*" - 2, because the edge *a *vanishes in the graph *L*(*G*). This equation ignores cases where edges in the graph *G*, *b *and *b*' for example, have a common node as endpoint (i.e., existence of triangles or *loops *in Fig. 3C). However, we can quantify these cases by using a new parameter *l*. As we can see in Fig. 3C–D, edges with one common node as endpoint in the graph *G *means one additional edge in the graph *L*(*G*). This additional edge in *L*(*G*) connects two neighbors of node *a*. By following definition of Eq. (1), it means that *n*_*a *_increases its value by one unit. We can consider these cases by increasing one unit the parameter *l *for each common node as endpoint of two edges in the graph *G *(for example, *l *= 1 means one common node). We write Eq. (4) after introducing the parameter *l *as:



where if *l *= 0 means that there are not *loops *and we recover Eq. (4). Though it is more realistic to consider the parameter *l *as a function of *k*' and *k*", we have considered *l *as an independent parameter. However, this simplification does not affect the qualitative features of our results. It should be noted that *l *always contributes to increasing the value of *C*_*a*_(*k*) and *C*_*a*_(*k*) ≤ 1 always holds from the definition. In order to study the limits of Eq. (5) we consider the following two cases:

• *a*) *k*' = *k*": We analyse the case where both degrees have the same value. We also consider the cases when *l *= 0 and *l *≠ 0 in order to study the effect of triangles. We show the results in Fig. 4. For large *k*', Eq. (5) goes asymptotically to 1/2 for *l *= 0 and *l *≠ 0. We also see that for *k*' ≥ 25, all lines are very close to 1/2. For low *k*' and *l *= 0, *C*_*a*_(*k*) takes values from 0.33 (*k*' = 3) to 0.48 (*k*' = 20). Hence, we see in Fig. 4 that higher values of *l *(more triangles) increase the values of *C*_*a*_(*k*).

• *b*) *k*" = constant, *k*' >>*k*": We plot in Fig. 5 three cases. *k*" is fixed with constant values as *k*" = 5 (black), *k*" = 10 (red), *k*" = 20 (blue) and *k*' is a free parameter. We see that *C*_*a*_(*k*) approaches to 1 when *k*' takes large values. For low *k*', the case *k*" = 5 shows a minimum with a few values of *k*' below 1/2. As we can see with dotted and dash-dotted lines in Fig. 5, the presence of triangles (*l *≠ 0) increases the value of *C*_*a*_(*k*). Finally, for *k*" = 10 and *k*" = 20, we see that only a few values of *C*_*a*_(*k*) are slightly below 1/2 for low *k*'. This analysis is complemented by calculating the minimum value of *C*_*a*_(*k*) analytically as: . The value of *k*', where the function *C*_*a*_(*k*) takes the minimum value, is given by:

where positive solution of the square root is written. By substituting this equation into Eq. (5), it is possible to calculate the minimum value of *C*_*a*_(*k*) for each configuration of *l *and *k*".

From these two cases, we can conclude that for hubs (i.e., those nodes with high degree (*k*' and *k*" >> 1)) and for highly clustered networks (many triangles *l *>> 1), the values of *C*_*a*_(*k*) in the transformed network are between around [, 1].

To calculate the distribution of *C*(*k*) in the transformed space (*C*^*T*^(*k*)) we introduce the concept of assortativity. By assortative (disassortative) mixing in networks we understand the preference for nodes with high degree to connect to other high (low) degree nodes [24]. By following *Newman *[24], we define the probability distribution to choose a randomly edge with two nodes at either end with degrees *k*' and *k*" as *e*_*k*'*k"*_. We also assume that the nodes of the initial network are following a power-law distribution *k*^-*γ *^and have no assortative mixing. Under these assumptions, the probability distribution *e*_*k*'*k" *_of edges that link together nodes with degree *k*' + *k*" can be written as:



We make a convolution between Eq. (4) and Eq. (7), by summing for all the possible degrees of the two nodes at either end of edges (*k*', *k*"), which can generate transformed nodes with degree *k *= *k*' + *k*" - 2. Thus, we obtain:



According to the structure of *C*^*T*^(*k*) and the behavior of *C*_*a*_(*k*) exposed above, *C*^*T*^(*k*) will grow smoothly for large *k*, i.e., scaling weakly with the node degree *k*. We have calculated numerically this expression and the results are discussed later in Fig. 7.

We have also calculated the analytical expression for <*C *>, and we have found that <*C *> has a size-independent behavior before and after the line graph transformation is done. We can write the number of nodes with degree *k *as:



and we assume that *C*(*k*) = *A*·*k *^-*α*^, where *A *is a constant. This constant changes when we consider hierarchical networks with different number of nodes in the initial cluster [7]. But it seems natural because in that case the degree distribution *P*(*k*) ~ *k*^-*γ *^of the network also changes. For <*C *> before the transformation we can write:



Note that the summation in the denominator begins with *k *= 1 because we renormalize over all the probability distribution.

Furthermore, we can obtain <*C *> by using the RSMOB model (explained in next section in detail). This model starts by generating a fully connected cluster of *m *nodes, such that the connectivity of each node is *k *= *m *- 1. In the following iteration, *m *- 1 replicas of the initial cluster are generated, and linked to the central node of the original cluster in such a way that the central node of the original cluster gains (*m *- 1)·(*m *- 1) edges, and its total connectivity being *k *= *m *- 1 + (*m *- 1)^2^. By iterating these procedure, it is easy to see that hubs (i.e., central nodes of each replica) will have connectivities , with *j *= 1, ..., log_*m*_*N *being the iteration number. Therefore, assuming that the degree distribution *P*(*k*) and the clustering coefficient *C*(*k*) are power-laws with exponents *γ*' and *α *respectively, the expression for <*C *> for the hubs reads as:



where *A*' is a constant adjusted so that <*C *>=1 holds for *j *= 1. The upper limit of the summation log_*m*_*N *is obtained by means of the expression *m*^*j *^= *N*, which gives the total number of nodes in the network and  denotes the exponent of the power-law distribution of hubs in the RSMOB model. We must note that in a hierarchical network, the number of nodes with different degree *k *is scarce, therefore the probability distribution of node degree is properly defined as *P*(*k*) = (1/*N*_*tot*_)(*N*_*k*_/Δ*k*), where *N*_*k *_is the number of nodes with degree *k*, *N*_*tot *_is the total number of nodes, and Δ*k *means that nodes with degree *k *are binned into intervals. In addition, we note that for the hierarchical model, Δ*k *changes linearly with *k*. Hence, the exponent of the power-law is given by *γ *= 1 + *γ*', with  where *m *is the number of nodes in the initial module.

By using Eqs. (10) and (11), we will see later (Tables 1 and 2) that <*C *> converges to a constant. In order to calculate <*C *> after the line graph transformation is applied (<*C*^*T *^>), we make the substitution *C*(*k*) → *C*^*T*^(*k*) in Eq. (10). As from Eq. (8) we have seen that *C*^*T*^(*k*) is almost constant, we can conclude that <*C*^*T *^> also has a constant behavior and it is almost independent with network size. While the scaling law of *C*(*k*) ~ *k*^-1 ^was proved mathematically in [6], here we have obtained the analytical expressions of *C*^*T*^(*k*), <*C *> and <*C*^*T *^>.

**Table 1:** Results of <*C *> evaluated by using Eq. (10) and the needed parameters in that calculation for 3 different setups: *γ *= 1 + *γ*', where  (*P*(*k*) ~ *k*^-*γ*^), *α *(*C*(*k*) ~ *k*^-*α*^), *A *(*C*(*k*) = *A*·*k*^-*α*^). Eq. (10) is a general expression of <*C *>.

*m *initial nodes	*γ*	*α*	*A*	<*C *>
3	2.58	1.1	2.34	0.20
4	2.26	1.1	3.68	0.36
5	2.16	1.1	5.18	0.54

**Table 2:** Results of <*C *> evaluated by using Eq. (11) for 3 different setups. The exponent of the power-law distribution of hubs is given by . The parameter *α *has same meaning as in Table 1. We also notice that in Eq. (11), *A*' is adjusted so that <*C *>=1 holds for *j *= 1. Eq. (11) is the particular expression of <*C *> applied to the RSMOB model.

*m *initial nodes	*γ*'	*α*	<*C *> (Eq. (11)
3	1.58	1.1	0.78
4	1.26	1.1	0.81
5	1.16	1.1	0.83

### Line graph transformation to a hierarchical network: numerical results

The RSMOB model [8] is able to reproduce the main topological features of the metabolic network. We follow the method described in [8] and generate a hierarchical network. Then, we apply the line graph transformation to that network.

**Figure 6:**
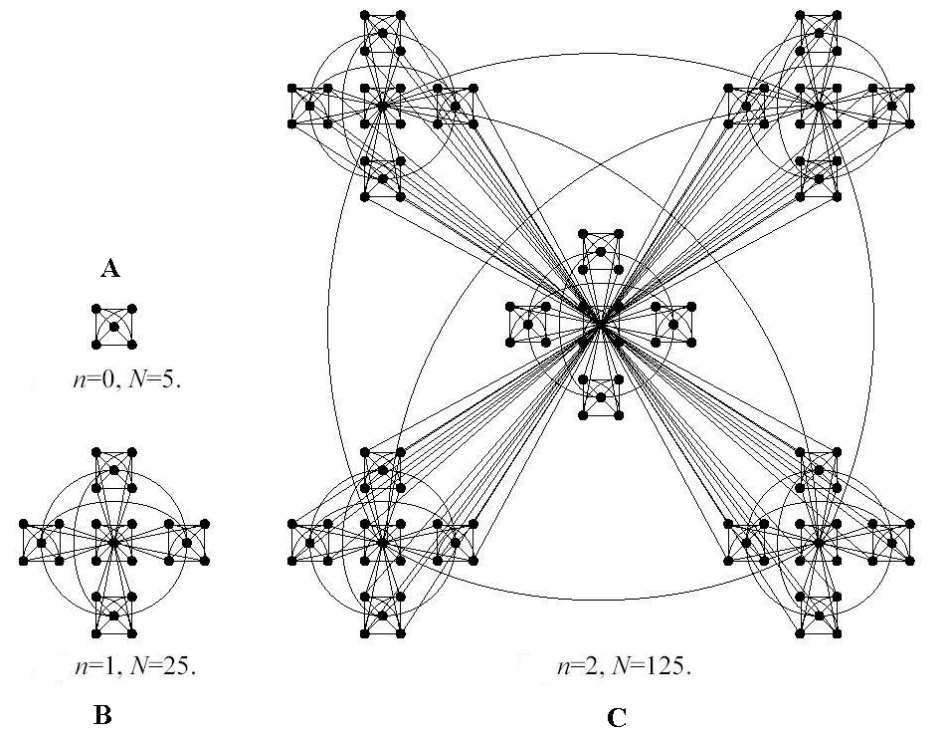
Hierarchical network generated by using the RSMOB model [8]. Starting from a fully connected cluster of 5 nodes, 4 identical replicas are created, obtaining a network of N = 25 nodes in the first iteration n = 1 (5^2 ^= 25 nodes). We have linked to each other the central hubs of the replicas by following [7]. This process can repeated indefinitely. We note that the initial number of nodes can be different than 5.

Fig. 6 illustrates the hierarchical network generated by the RSMOB model. The network is made of densely linked 5-node modules (it is worth noticing that the number of nodes in the initial module can be different than 5) that are assembled into larger 25-node modules (iteration n = 1, 5^2 ^= 25 nodes). In the next step four replicas are created and the peripheral nodes are connected again to produce 125-node modules (iteration n = 2, 5^3 ^= 125 nodes). This process can be repeated indefinitely [8].

**Figure 7:**
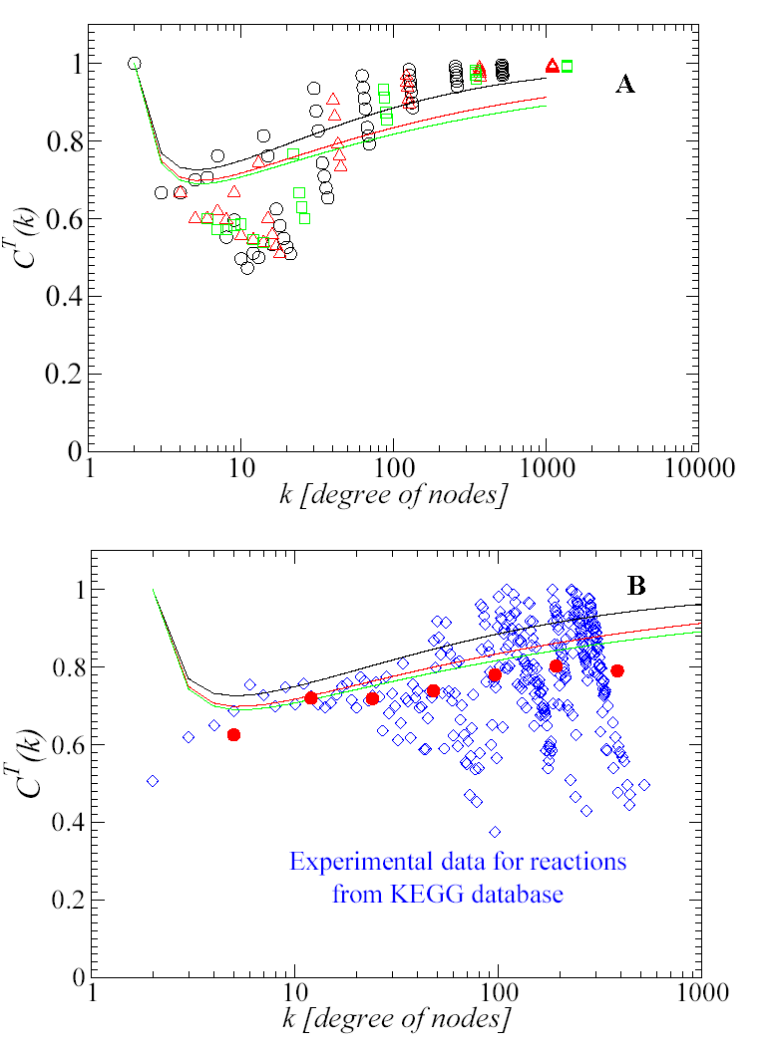
(A) We plot the results of the hierarchical model for *C*^*T*^(*k*) for different configurations. 3 initial nodes and up to 7 iterations (circles), 4 initial nodes and up to 5 iterations (triangles), 5 initial nodes and up to 4 iterations (squares). Prom top to bottom (3 initial nodes (black), 4 initial nodes (red), 5 initial nodes (green)), we show with lines the results of *C*^*T*^(*k*) obtained by means of Eq. (8). (B) The lines have the same meaning as before and the diamonds correspond to the experimental data for reactions from the KEGG database [14]. Experimental data involves 163 organisms. Circles (red): Experimental data binned into seven intervals according to degree (1 <*k *≤ 8 <, ..., 128 <*k *≤ 256, 256 <*k *≤ 512). Figures in log-linear scale.

To evaluate *C*(*k*), we have constructed three hierarchical networks with 3, 4, and 5 initial number of nodes. These networks were generated up to 7 (6561), 5 (4096), and 4 (3125) iterations (nodes), repectively. Once we have constructed these three networks, we apply the line graph transformation to them, and we calculate the *C*^*T*^(*k*) clustering coefficient for the transformed networks. In Fig. 7A we show the results of the clustering coefficient of the transformed network. Circles, triangles and squares indicate the values of *C*^*T*^(*k*) for the transformed network with 3, 4, and 5 initial nodes, respectively. In Fig. 7A we also plot with continuous lines the values of *C*^*T*^(*k*) obtained from Eq. (8). From top to bottom the lines correspond to the networks of 3, 4 and 5 initial nodes, respectively. In Fig. 7A, we see that the lines show an acceptable agreement with the overall tendency of data generated by the transformed network. In Fig. 7B, we see that the results from theoretical calculation of *C*^*T*^(*k*) via Eq. (8) (lines) are in good agreement with the experimental data (diamonds) from the KEGG database [14]. Moreover, in order to have enough statistics to compare with the analytical expression for the *C*^*T*^(*k*), we have binned into seven intervals the experimental data according to degree *k *(1 <*k *≤ 8 < ,..., 128 <*k *≤ 256, 256 <*k *≤ 512), and averaged over the *C*^*T*^(*k*)'s obtained in that range (red circles). It shows a better agreement between KEGG results and the analytical curves. The only disagreement comes at *k *= 2. This is easy to understand because in the hierarchical model depicted in Fig. 6, we can only find *C*(*k *= 2) = 1 for 3 initial nodes by construction of the network. However, in real networks, we could find nodes which have only two neighbors and, in some cases, these neighbors could be connected. In these cases the clustering coefficient takes value one.

**Figure 8:**
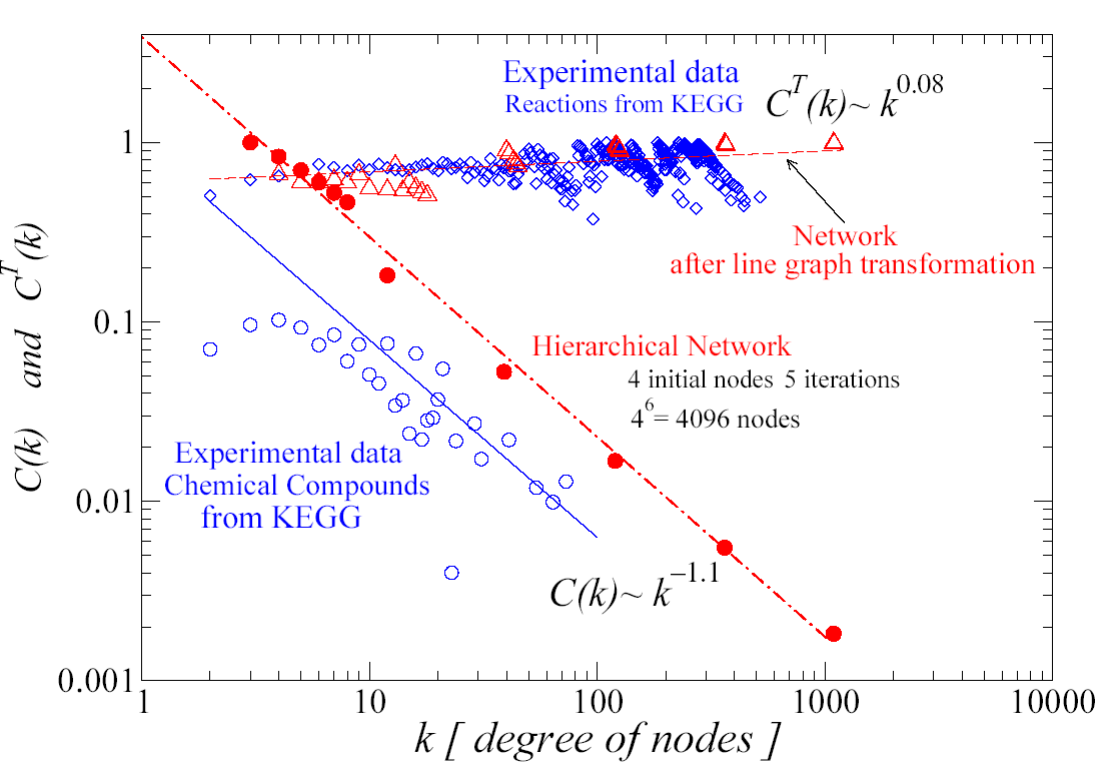
Full circles (red) and dot-dashed line (red): *C*(*k*) evaluated with the hierarchical network. Empty triangles (red) and dashed line (red): *C*(*k*) after the line graph transformation is done over the hierarchical network (*C*^*T*^(*k*)). Diamonds (blue): *C*^*T*^(*k*) of reactions data from the KEGG database [14]. Empty circles (blue) and continuous line: *C*(*k*) of compounds data from KEGG. Hierarchical model with 4 initial nodes and 5 iterations. Figure in log-log scale.

In Fig. 8, we show the results for *C*^*T*^(*k*) after the line graph transformation is applied to the hierarchical network generated by 4 initial nodes and up to 5 iterations. The results are shown with empty triangles (red) and fitted to the dashed line. We see that *C*(*k*) ~ *k*^-1.1 ^changed into *C*^*T*^(*k*) ~ *k*^0.08^. We also see that the line graph transformation increases the average of the clustering value of the transformed network. These theoretical results were compared with the experimental data from KEGG [14], finding a good agreement, and supporting the result of a degree-independent clustering coefficient *C*^*T*^(*k*) for the reaction network.

**Figure 9:**
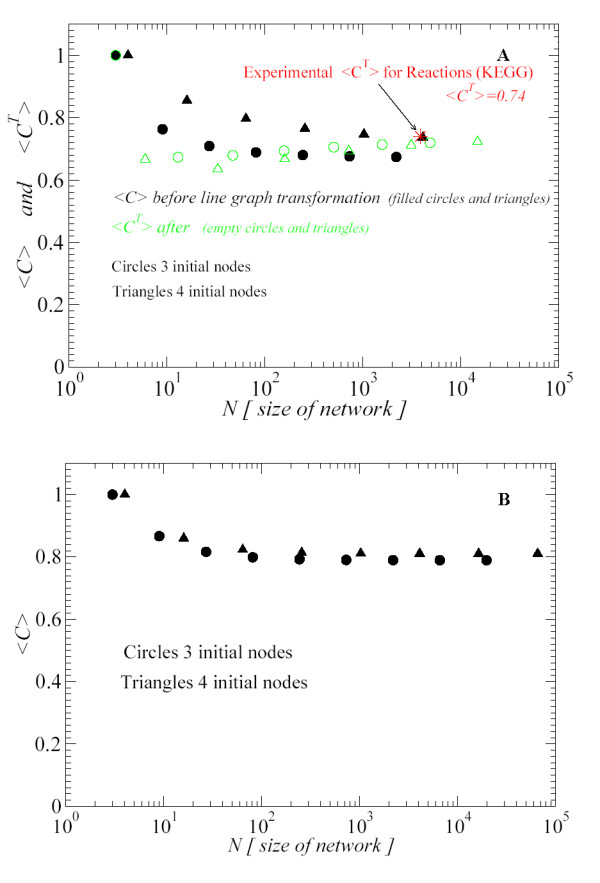
Dark (black): <*C *> is calculated by using the hierarchical network. Light (green): <*C*^*T *^> (<*C *> after the line graph transformation is applied to the hierarchical network). Circles (3 initial nodes), Triangles (4 initial nodes). Star (red): Experimental <*C*^*T *^> for reactions from the KEGG database [14]. (B) <*C *> is calculated by using Eq. (11). The results show a good agreement and similar tendency to those shown in Fig. 9(a) (dark circles and triangles). Figures in log-linear scale.

For <*C *> we have evaluated Eq. (10) for 3 different configurations. We have considered 3 initial nodes, 4 and 5 initial nodes nodes up to 7, 5 and 4 iterations, respectively. As it is explained in [7], <*C *> approaches asymptotically to a constant value, being independent of the size of the network. The asymptotic value depends on the initial number of nodes. We calculated the values of *γ *corresponding to the degree distribution *P*(*k*) ~ *k*^-*γ *^for each network, and the related constant *A*, which appears in Eq. (10). We show in Table 1 the values of these parameters and the results of <*C *> obtained by Eq. (10). These values, as it can be seen in Fig. 9A, are below the asymptotic values of ~ 0.66 (circles) and ~ 0.74 (triangles) obtained by using the RSMOB model. However, we have found an explanation for this result. In Fig. 8, the full circles at the top of the dash-dotted line correspond to non-hubs nodes. We have checked that these nodes do not follow a power-law, hence the value of *C*(*k*) is being overestimated by the scaling dependence *k*^-1 ^and it provides a larger value of <*C *>. In [7], the values of <*C *> from hierarchical model were compared with the experimental values of 43 organisms. The values of <*C *> for each organism were around 0.15 – 0.25. By using the KEGG database we have evaluated the experimental value <*C *> for 163 organisms and we obtained an average value of 0.08.

We show in Fig. 9A the values of <*C *> calculated for networks generated by 3 initial nodes (circles) and 4 initial nodes (triangles) by using the RSMOB model. We see that <*C *> approaches asymptotically to constant values around ~ 0.66 (circles) and ~ 0.74 (triangles), being independent of the size of the network. Once the line graph transformation is applied, we see that the corresponding values of <*C*^*T *^> also approach asymptotically to constant values. Hence, <*C*^*T *^> also is size-independent for large *N *(empty circles and triangles). In addition, we have averaged the experimental value of the clustering coefficient for reactions of 163 organisms found in KEGG database and we have obtained the value of <*C*^*T *^>= 0.74. We see that the experimental value <*C*^*T *^> for reactions is in good agreement with the asymptotic values obtained by the transformed network (empty triangles and circles).

Furthermore, we have also calculated <*C *> by using Eq. (11). This equation should reproduce the results of <*C *> calculated by using the RSMOB model (dark circles and triangles in Fig. 9A). In Fig. 9B, we see that the results are qualitatively similar to those shown in Fig. 9A (dark circles and triangles).

We remark that the theoretical analysis of <*C *> and <*C*^*T *^> done here has also been useful to prove that they are independent of network size.

**Figure 10:**
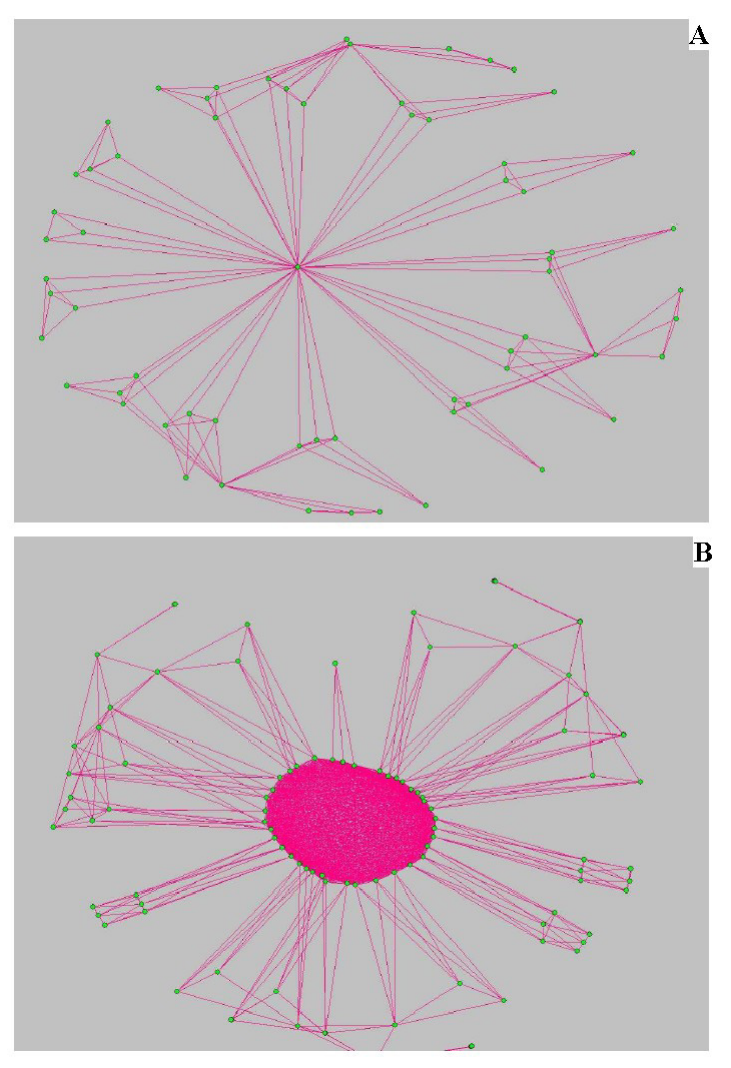
(A) Hierarchical network generated by using the model of ref. [8] with 4-node modules and up to 2 iterations. (B) Network after the line graph transformation. We see a huge interlinked cluster in the center of figure, which generates the degree-independent clustering coefficient *C*^*T*^(*k*) (it scales weakly as *C*^*T*^(*k*) ~ *k*^0.08^).

Finally, in Fig. 10 we plot the hierarchical network (left) and the transformed network (right) by using the graph drawing tool *Pajek *[25]. We see the high degree of compactness of the transformed network. It could be related to the concept of robustness of a network. It means that by removing one node randomly from the reaction network depicted in the Fig. 10, the normal behavior of the cell might be preserved by finding an alternative path (reaction) to complete the task. This fact could be a consequence of the high degree of clustering and connectivity between the nodes in the transformed network.

## Conclusions

We have studied here the clustering coefficients *C*(*k*) and <*C *> of the reaction network by applying the line graph transformation to a hierarchical network. This hierarchical network was generated by using the RSMOB model, which reproduces properly the topological features of the metabolic network, in particular the compound network. Our results indicate that by applying the line graph transformation to the hierarchical network, it is possible to extract topological properties of the reaction network, which is embedded in the metabolic network. The RSMOB model stores the adequate information of the reaction network and the line graph transformation is one useful technique to evoke it.

While *C*(*k*) scales as *k*^-1.1 ^for the initial hierarchical network (compound network), we find *C*(*k*) ~ *k*^0.08 ^for the transformed network (reaction network). This theoretical prediction was compared with the experimental data from the KEGG database, finding a good agreement. Our results indicate that the reaction network is a degree-independent clustering network. Furthermore, the weak scaling of *C*(*k*) for the reaction network suggests us that this network may not have hierarchical organization. However, further analyses of this network, and in general of all biological networks, by following the concept of hierarchical path are encouraged [20,21].

On the other hand, we have also conducted an analytical derivation for the clustering coefficients *C*(*k*) and <*C *>. Expressions for these coefficients were calculated before and after the line graph transformation is applied to the hierarchical network. The agreement obtained by using these expressions was found acceptable, and consequently, they could be useful for further analyses in different networks (biological and non-biological).

The line graph transformation has recently been applied on metabolic networks [13] to study the scale-free topology of the reaction network, and on the protein-protein interaction network to detect functional clusters [15]. The work done here is another important application of this interesting technique.

## Authors' contributions

JCN conceived of the study, designed and implemented the analyses, and prepared the manuscript. NU carried out computational implementations and experiments. TY participated in the acquisition and processing of data from KEGG database. MK provided conceptual guidance and data from the KEGG database, and conceived the initial idea of the two complementary metabolic networks. TA provided guidance, coordinated and participated in the biological and theoretical analyses, and revised the manuscript. All authors read and approved the final manuscript.

**Table 3:** Definitions of functions and their values before and after the line graph transformation is applied to the hierarchical network. *N*_*k*_: number of nodes of degree *k*. The † symbol means that these dependences were analyzed in the present work, while the * symbol means that it was studied in our previous work [13].

Func.	Definition	Dependence *before*	Dependence *after *(Eq. (11)
*P*(*k*)	*N*_*k*_/*N*	*k*^-*γ*^	*k*^-*γ*+1 ^*
*C*_*i*_(*k*)	2*n*/[*k*_*i*_(*k*_*i *_- 1)]	*k*^-1.1^	*k*^0.08†^
<*C *>		size-independent^†^	size-independent^†^
